# PI-HydroGNN: a physics-informed spatiotemporal graph neural network framework for hydraulic reliability, leakage detection, and energy-efficient operation in water distribution systems

**DOI:** 10.1038/s41598-026-57290-y

**Published:** 2026-06-18

**Authors:** Jayaprakash Chinnadurai, KarthiPrem S, Sangeetha Ramaswamy, Sundara Kumar M. R, Sandeep Kumar Mathivanan, Sangeetha S. K.B

**Affiliations:** 1 School of Computing Science and Engineering, Galgotias University, Greater Noida, 203201 India; 2 Department of Computer Science and Engineering, Shree Sathyam College of Engineering and Technology, Sankari, Salem, Tamilnadu India; 3 School of Computing, Department of Computer Science and Engineering, VelTech Rangarajan Dr sagunthala R&D Institute of Science and Technology, Chennai, India; 4 Department of CSE (AI & DS), Dayananda Sagar University, Harohalli, Bengaluru, India; 5 Department of Computer Science and Engineering- AI & ML, KG Reddy College of Engineering and Technology, Hyderabad, Telangana 501504 India; 6 Manipal Institute of Technology Bengaluru, Manipal Academy of Higher Education, Manipal, India

**Keywords:** Energy science and technology, Engineering, Mathematics and computing

## Abstract

For Water Distribution Networks (WDS) to be sustainably operated, hydraulic reliability, leak detection, and energy-efficient operation are essential. Although the data-driven hydraulic modeling problem has been advanced by recent developments in Graph Neural Networks (GNNs), the existing solutions are black-box predictors that lack the ability to directly enforce the physical conservation laws, which makes them less robust, interpretable, and reliably performing. The research proposes PI-HydroGNN, a physics-informed spatiotemporal graph learning framework for water distribution system monitoring and control. The framework integrates established modeling components within a unified architecture and incorporates hydraulic mass balance, energy conservation, and pressure-dependent leakage dynamics directly into the optimization objective, improving physical consistency and robustness. Three benchmark networks (Net3, C-Town, and Anytown) with stochastic demand variations and artificial leakage scenarios over a 60-day horizon are used in the extended period EPANET simulations to evaluate the framework. Compared to the data-driven baselines, PI-HydroGNN achieves 9.6% savings in pump energy use, an F1-score improvement of 0.922 for leakage detection, and a 39.9% reduction in pressure prediction RMSE, while also decreasing pressure violation rates by 68.9%. The model demonstrates strong generalization across benchmark networks under varying demand and leakage conditions. The findings show that a high-quality and functionally well-integrated digital twin for WDS operation can be obtained by incorporating physical principles into graph-based learning. PI-HydroGNN demonstrates strong potential for digital twin applications in water distribution system management.

## Introduction

To address the drawbacks of the traditional hydraulic solvers in dynamic conditions, Water Distribution Systems (WDS) are currently being modeled using physics-informed and graph-based learning paradigms. To enhance the reliability of predictions, a Physics-Informed Graph Neural Network (PI-GNN) for hydraulic state estimation in WDS is introduced in ^1^, which integrates the physics equations of flow into graph-based models. ^2^ introduces a Spatio-Temporal Graph Physics-Informed Neural Network (ST-GPINN) for water quality prediction, which integrates temporal learning and hydraulic equations to enhance the reliability of chlorine concentration predictions. ^3^ emphasizes robust and scalable PI-GNN models that can effectively deal with large water distribution systems with enhanced computing efficiency.

The idea of physics-informed spatio-temporal graph learning has been used in subsurface and reservoir phenomena, apart from urban water supply. The applicability of physics-informed graph learning in fluid-driven phenomena is demonstrated in ^4^, where a physics-informed spatio-temporal GNN is proposed for water flood management. The need to incorporate physical residuals in the optimization process is emphasized in ^5^, which further improves physics-informed gradients for the solution of practical WDS problems. To improve the reliability of predictions and explainability, ^6^ combines hydraulic simulation models with GNNs that are physics and chemistry informed for chlorine transport and decay.

Recent studies also investigate graph designs for transformer architectures. ^7^ introduce a physics-informed graph transformer fusion model for leakage detection and grading, incorporating hydraulic physics and attention mechanisms. The generalization ability of physics-informed GNNs is shown in ^8^ for nonlinear fluid flow prediction in porous media problems. The scalability of physics integration with graph structures is shown in ^9^ for spatiotemporal production forecasting in greenery problems using physics-informed GNNs. Power and energy distribution grids also increasingly apply physics-informed education. In ^11^, physics-informed multi-agent deep reinforcement learning is applied for pump schedule optimization in urban water supply stations, while in ^10^, physics-informed GNNs are applied for voltage regulation in unbalanced distribution grids.

For fluid flow modeling in a marine setting, hybrid data-physics modeling approaches such as ^12^, which integrates graph convolutional networks with physical constraints, is employed. In ^13^, a comprehensive hybrid approach for wastewater resilience is introduced, integrating physics-informed models and graph AI to demonstrate the full system modeling potential. As discussed in ^14^, disaster defense using a unified framework that integrates physical simulations with AI, digital twin concepts have further advanced physics-informed AI for flood prediction and mitigation. In ^15^, the application of supervised and semi-supervised GNNs for state estimation in WDS is explored, demonstrating graph learning as a productive alternative to conventional state estimators.

The development of physics-informed neural networks for water and wastewater networks is discussed in ^16^ to overcome the issues of scalability, uncertainty quantification, and real-time implementation. The multi-level knowledge integration is demonstrated in ^17^ by proposing hierarchical physics-informed neural networks that incorporate transfer learning for contamination simulation. The usability of physics-embedded graph learning is demonstrated by its applications in the energy and environmental sectors. ^19^ explores the application of physics-informed neural networks in power systems, concentrating on constraint satisfaction and stability analysis, ^18^ presents hybrid GNN-PINN models for subsurface soil temperature simulation. In ^20^, a benchmarking task with defined evaluation procedures for chlorine status estimation in WDS is proposed.

In ^21^, physics-informed machine learning is used to demonstrate universal modeling for water quality parameters, outperforming other generalization abilities for different demand scenarios. The relevance of topology-informed learning with physics-based connectivity has been understood by the application of physics-embedded graph learning to basin-scale river network modeling ^23^ and integrated energy systems ^22^. Ref ^24^ presents the application of physics-informed neural networks for predictive control of district heating systems, with emphasis on the incorporation of physics laws into control optimization problems.

Despite the advances, the present research is mainly concerned with energy optimization, leak detection, water quality forecasting, hydraulic state estimation separately. Few studies provide a comprehensive physics-informed graph learning method that address hydraulic mass balance, energy optimization, leak detection, and pump schedule optimization simultaneously. Despite the application of reinforcement learning to pump scheduling ^11^, there is limited progress in the incorporation of reinforcement learning into physics-regularized graph learning with strong hydraulic feasibility constraints.

Table 1 depicts the summary of recent studies (2023–2025) on water quality prediction and Water Quality Index (WQI) modeling, highlighting study focus, methodologies employed (e.g., LSTM-based deep learning, LightGBM, XGBoost, Random Forest with SHAP), and identified research gaps related to mechanistic interpretability, scalability, and limited physics integration.

**Table 1:** Comparative analysis of traditional approaches for water quality prediction and identified research gaps.

References	Study focus	Methods used	Gap/limitation identified
^25^	Continental-scale water quality prediction & model reliability	LSTM-based deep learning with uncertainty analysis	Limited mechanistic interpretability; scalability concerns
^26^	Water Quality Index (WQI) prediction	LightGBM, XGBoost, Random Forest + SHAP	Primarily data-driven; lacks physics integration
^27^	Drinking water classification	ANN, SVM, ensemble ML	Limited generalization across regions
^28^	Review of DL applications in water systems	CNN, LSTM, GNN overview	Highlights lack of interpretability & physical consistency
^29^	AutoML-based WQI modeling	AutoML pipeline + hyperparameter optimization	Black-box modeling; limited uncertainty quantification
^30^	Real-time monitoring with IoT sensors	RF, ANN integrated with sensor networks	Limited deep learning comparison
^31^	Spatiotemporal water parameter modeling	CNN-LSTM hybrid model	Computational complexity & interpretability issues

For time-series forecasting and anomaly detection in infrastructure systems, recent research has investigated purely data-driven models, such as transformer-based architectures and probabilistic learning frameworks, in addition to physics-informed methods^32–34^. Strong predictive performance and flexibility are provided by these techniques, but they could not be robust or physically consistent under unknown operating situations. For operational decision assistance, hybrid industrial systems also include data analytics and simulation models. To balance interpretability and accuracy, the suggested system integrates data-driven learning with physical restrictions.

Previous research has generally used either physics-based or data-driven methods to address hydraulic state estimates, leak detection, and pump control as distinct issues. This study suggests a unified architecture that combines these tasks into a single spatiotemporal graph neural network with a reinforcement learning module that is guided by physics. A major shortcoming in the analytics of the current water distribution system is addressed by this joint approach, which permits coordinated monitoring and control under stochastic demand situations.

The proposed PI-HydroGNN framework overcomes these challenges by designing a new Physics-Informed Graph Neural Network that incorporates reinforcement learning for energy-efficient pump operation and directly incorporates the hydraulic governing equations into a spatial–temporal graph learning model. To further improve intelligent and scalable water infrastructure management, the proposed framework seeks to accomplish real-time hydraulic status estimation, precise leakage detection, and energy-efficient control with uncertain demand.

## Proposed PI-HydroGNN framework

### Problem formulation

Consider a directed graph G = (V,E) that represents a water distribution system. In this graph, V stands for the collection of nodes that represent junctions, reservoirs, and tanks, whereas E denotes represents the set of edges that represent pipes and pumps. Every node i ∈ V is linked to hydraulic state variables at each time step t, such as demand D_i_ (t), hydraulic head H_i_ (t), and pressure P_i_ (t). The flow rate Q_ij_ (t), hydraulic resistance parameters, and the operating condition of the pump are linked to each edge (i,j) ∈ E. Under dynamic demand conditions, PI-HydroGNN aims to collaboratively carry out hydraulic state estimation, leakage localization, and energy-efficient pump scheduling while guaranteeing adherence to the relevant hydraulic rules.

Formally, the model seeks to learn a mapping:

1 $$\mathcal{F}:\{G,{X}_{t-k:t},{E}_{t-k:t}\}\to \{{\widehat{P}}_{t+1},{\widehat{L}}_{t},{\widehat{A}}_{t}\}$$

where ${X}_{t-k:t}$ represents historical node features over a temporal window of length *k*, ${E}_{t-k:t}$ denotes edge features, ${\widehat{P}}_{t+1}$ is the predicted future pressure state, ${\widehat{L}}_{t}$ represents leakage predictions, and ${\widehat{A}}_{t}$ denotes optimal pump control actions.

### Spatio-temporal architecture

#### Graph representation and spatial learning

To capture spatial dependencies across the hydraulic network, a Graph Convolutional Network (GCN) is employed. At each time step *t*, the node feature matrix is defined as:

2 $${X}_{t}\in {\mathbb{R}}^{N\times F}$$

where *N* is the number of nodes and *F* represents node feature dimensions (pressure, head, demand, tank level). The adjacent matrix *A* encodes the network connectivity derived from the pipe and pump topology.

The spatial message passing operation is defined as:

3 $${H}_{t}^{\left(l+1\right)}=\sigma \left({\widetilde{D}}^{-1/2}\widetilde{A}{\widetilde{D}}^{-1/2}{H}_{t}^{\left(l\right)}{W}^{\left(l\right)}\right)$$

where $\widetilde{A}=A+I$ includes self-connections, $\widetilde{D}$ is the degree matrix, ${W}^{\left(l\right)}$ are learnable weights, and $\sigma (\cdot )$ is a nonlinear activation function. This operation enables the model to propagate hydraulic information across connected nodes, reflecting physical flow interdependence.

#### Temporal learning with TCN

While GCN captures spatial correlations, temporal dynamics arising from demand fluctuations and operational changes are modeled using a Temporal Convolutional Network (TCN). For each node, a historical window of length *k* is constructed:

4 $${\mathcal{X}}_{t}=[{X}_{t-k},\dots ,{X}_{t}]$$

The TCN applies dilated causal convolutions to learn long-range temporal dependencies:

5 $${Z}_{t}=\sum_{i=0}^{k}{w}_{i}\cdot {X}_{t-i}$$

where dilation allows exponential receptive field growth without increasing model depth excessively. This structure enables the network to capture daily demand cycles, delayed hydraulic responses, and cumulative effects of pump operations.

#### Integrated spatio-temporal representation

The outputs of spatial and temporal modules are fused through concatenation followed by nonlinear transformation:

6 $${F}_{t}=\phi ([{H}_{t}^{\mathrm{GCN}}{\hspace{0.17em}}\mid \mid {\hspace{0.17em}}{Z}_{t}^{\mathrm{TCN}}])$$

where $\mid \mid$ denotes concatenation and $\phi (\cdot )$ represents a fully connected transformation. The fused representation ${F}_{t}$ serves as the shared embedding for downstream prediction tasks.

### Physics-informed loss formulation

To ensure physical consistency, hydraulic governing laws are embedded directly into the learning objective.

#### Mass conservation constraint

At each junction node *i*, the continuity equation requires that inflow minus outflow equals demand and leakage:

7 $$\sum_{j}{Q}_{ji}(t)-\sum_{j}{Q}_{ij}(t)={D}_{i}(t)+{Q}_{leak,i}(t)$$

The mass balance loss is defined as:

8 $${L}_{mass}=\frac{1}{N}\sum_{i=1}^{N}\mid \sum_{j}{Q}_{ji}-\sum_{j}{Q}_{ij}-{D}_{i}\mid$$

#### Energy (head-loss) constraint

For each pipe $\left(i,j\right)$, the Hazen–Williams equation governs head loss:

9 $${H}_{i}-{H}_{j}={R}_{ij}\mid {Q}_{ij}{\mid }^{1.852}$$

Deviation from this relation is penalized via:

10 $${L}_{energy}=\frac{1}{\mid E\mid }\sum_{(i,j)\in E}\mid ({H}_{i}-{H}_{j})-{R}_{ij}\mid {Q}_{ij}{\mid }^{1.852}\mid$$

#### Leakage regularization

Leakage-induced pressure deviations are captured through:

11 $${L}_{leak}=\frac{1}{N}\sum_{i=1}^{N}\mid {\widehat{P}}_{i}-{P}_{i}\mid \cdot {1}_{\mathrm{leak}}$$

where ${1}_{\mathrm{leak}}$ indicates leak presence.

The overall loss function combines supervised data loss and physics constraints:

12 $${L}_{total}={L}_{data}+{\lambda}_{1}{L}_{mass}+{\lambda}_{2}{L}_{energy}+{\lambda}_{3}{L}_{leak}$$

where ${\lambda}_{1},{\lambda}_{2},{\lambda}_{3}$ are balancing hyperparameters.

This formulation ensures that predictions remain hydraulically feasible while maintaining predictive accuracy.

### Reinforcement learning pump control module

To minimize operational energy consumption while maintaining pressure constraints, pump scheduling is formulated as a constrained Markov Decision Process.

The system state at time *t* is defined as:

13 $${s}_{t}=\{{P}_{t},{H}_{t},\text{tank levels},\text{pump status}\}$$

The action space consists of discrete pump ON/OFF commands or continuous speed adjustments. The reward function is defined as:

14 $${r}_{t}=-\left({C}_{energy}(t)+\alpha \cdot {\mathrm{PressureViolation}}(t)\right)$$

where ${C}_{energy}(t)$ denotes energy cost and the second term penalizes pressure violations.

Policy optimization is performed using deep reinforcement learning to learn an optimal control strategy:

15 $${\pi }^{*}=\mathrm{a}\mathrm{r}\mathrm{g}\underset{\pi }{\mathrm{m}\mathrm{a}\mathrm{x}}{\mathbb{E}}\left[\sum_{t}{\gamma }^{t}{r}_{t}\right]$$

The RL agent leverages PI-HydroGNN predictions as state inputs, enabling proactive and energy-aware decision-making.

16 $${\mathrm{L}}_{{{\mathrm{data}}}} = \lambda_{{1}} {\mathrm{L}}_{{{\mathrm{reg}}}} + \lambda_{{2}} {\mathrm{L}}_{{{\mathrm{cls}}}}$$

The data fidelity loss ${L}_{\mathrm{data}}$ is formulated as a combination of regression and classification losses to support the multi-task learning objective. The regression component ${L}_{\mathrm{reg}}$ is defined using Mean Squared Error (MSE) for continuous variables such as nodal pressure:

17 $${L}_{\mathrm{reg}}=\frac{1}{N}\sum_{i=1}^{N}({y}_{i}-{\widehat{y}}_{i}{)}^{2}$$

where ${y}_{i}$ and ${\widehat{y}}_{i}$ denote the true and predicted pressure values, respectively.

The classification component ${L}_{\mathrm{cls}}$ is defined using cross-entropy loss for leakage detection and severity classification tasks:

18 $${L}_{\mathrm{cls}}=-\frac{1}{N}\sum_{i=1}^{N}{y}_{i}\mathrm{l}\mathrm{o}\mathrm{g}({\widehat{y}}_{i})$$

where ${y}_{i}$ represents the ground truth class label and ${\widehat{y}}_{i}$ is the predicted probability.

The overall data loss is computed as a weighted sum of these components, where ${\lambda}_{1}$ and ${\lambda}_{2}$ are hyperparameters that balance regression and classification objectives. This data fidelity term is combined with the physics-based loss components to form the total training objective.

### Optimization and training strategy

Mini-batch gradient descent with the Adam optimizer is employed for training the model. Sliding windows are utilized for forming temporal sequences, and only the training data is used for calculating normalization statistics. For stable convergence, physics regularization terms are gradually added during the first training epoch. Overfitting is prevented by early stopping based on validation loss. The proposed PI-HydroGNN system integrates reinforcement learning-based pump control, physics-constrained optimization, temporal convolutional modeling, and spatial graph learning into unified architecture. Compared to data-driven approaches, the framework provides improved generalization, physical realizability, and efficiency by directly incorporating the hydraulic governing equations into the learning objective.

## Experimental protocol and evaluation metrics

### Dataset construction and realistic simulation framework

The benchmark water distribution networks used in the U.S. Environmental Protection Agency’s (EPA) EPANET 2.2 hydraulic simulation environment were employed to generate the dataset used to evaluate the proposed PI-HydroGNN framework. To enable automatic simulation-learning interaction, a PyTorch-based deep learning pipeline was integrated with the EPANET toolkit interface. Before experimentation, all networks were retrieved in the standard EPANET.inp format and checked for hydraulic correctness, including stability of convergence during extended period simulation, demand pattern integrity checks, and connectivity checks.

#### Network selection and structural characteristics

Three widely adopted benchmark networks were selected to ensure structural diversity, scalability testing, and task-specific suitability:

- Net3 has 3 tanks, 2 pumps, 117 pipes, and 92 nodes. Due to its well-connected topology and low complexity, this medium-scale network was selected for hydraulic state estimation analyses.
- C-Town has11 pumps, 7 tanks, 429 pipes, and 388 nodes. Due to its true operational diversity and range, this large-scale network was selected for leakage detection analyses.
- Anytown has three pumps, one storage tank, 25 nodes, and 44 pipes. Energy-efficient pump scheduling optimization was performed using this pump-dense but smaller network.

A directed graph G = (V,E) was employed to model each network, with nodes representing junctions, tanks, and reservoirs, and edges representing pipes and pumps. Although dynamic reversal was allowed during simulation, directionality was assigned based on the nominal direction of flow.

#### Hydraulic simulation configuration

With a hydraulic time, step and update interval of 1-h, extended period simulations were performed over a continuous period of 60 days, resulting in 1440 temporal instances for each network. Unlike demand-driven models, the Pressure-Driven Demand (PDD) model was employed to ensure realistic demand reduction under low-pressure conditions. The Hazen-Williams formula was employed to determine head loss while preserving network-specific values of the roughness coefficient, as specified in the original benchmark models. To ensure convergence, the hydraulic solver tolerance was specified to be 0.001 m, with a maximum of 40 trials per time step. The pump curves and minor losses were preserved exactly as in the original models. At each hourly time step, the hydraulic variables were extracted which is shown in Table 2.This configuration ensured sufficient temporal depth for spatio-temporal learning while preserving hydraulic realism.

**Table 2:** Hydraulic variables extracted at node, edge, and system levels during extended period simulation.

Level	Variable name	Symbol	Unit	Description
Node	Pressure	P_i_	m	Nodal pressure head relative to local ground elevation at junction or tank i
Node	Hydraulic head	H_i_	m	Total hydraulic head including elevation and pressure components at node i
Node	Actual delivered demand	D_i_	L/s	Pressure-driven water demand supplied at junction i
Node	Tank water level	L_i_	m	Water surface elevation within storage tank i above tank bottom
Node	Leak discharge	Q_leak,i_	L/s	Pressure-dependent leakage outflow at node i, activated when emitter coefficient is non-zero
Edge	Pipe flow rate	Q_ij_	L/s	Volumetric flow rate in pipe connecting nodes i and j
Edge	Flow direction	F_ij_	–	Binary indicator representing nominal flow orientation (1 = forward, 0 = reverse)
Edge	Head-loss	h_f,ij_	m	Energy loss due to friction along pipe or pump between nodes i and j
Edge	Pump operational state	S_p_	–	Pump activation state (binary ON/OFF) or normalized speed ratio when variable speed control is applied
System	Total network demand	D_total_	L/s	Aggregate delivered demand across all junction nodes at time t
System	Total pump energy consumption	E_total_	kWh	Cumulative electrical energy consumed by all pumps during time interval t
System	Pressure violation indicator	V_p_	–	Binary variable indicating violation of minimum pressure constraint at any monitored node

Water distribution systems include additional challenges such sensor noise, missing measurements, parameter uncertainty, and operational variability, even though EPANET-based simulations provide a controlled and repeatable evaluation environment. Stochastic demand perturbations, pressure-dependent leakage models, and controlled noise injection were included to the simulation framework to close this gap and simulate realistic field conditions. Water distribution systems present additional challenges, such as sensor noise, missing readings, unclear pipe characteristics, and operational uncertainty, whereas EPANET-based simulations offer a controlled and repeatable evaluation environment. The simulation approach includes controlled noise injection, pressure-dependent leakage modeling, and stochastic demand perturbations to close this gap. These improvements seek to preserve experimental repeatability while approximating uncertainty.

#### Stochastic demand modeling

To emulate realistic urban consumption dynamics, baseline nodal demands were perturbed using a multiplicative stochastic model. For each junction *i* at time *t*, demand was defined as:

19 $${D}_{i,t}={D}_{i,base}\cdot {\phi}_{t}\cdot (1+{\epsilon}_{i,t})$$

where ${\phi}_{t}$ represents deterministic diurnal multipliers, ${\epsilon}_{i,t}\sim \mathcal{N}(0,{\sigma }^{2})$ is Gaussian noise.

The standard deviation *σ* was varied between 0.05 and 0.15 to represent 5–15% stochastic variability across experiments. Diurnal demand multipliers used for stochastic consumption modeling. Independent random seeds were assigned to training, validation, and testing splits to prevent temporal leakage and ensure that evaluation scenarios contained unseen stochastic demand realizations.

**Fig. 1:**
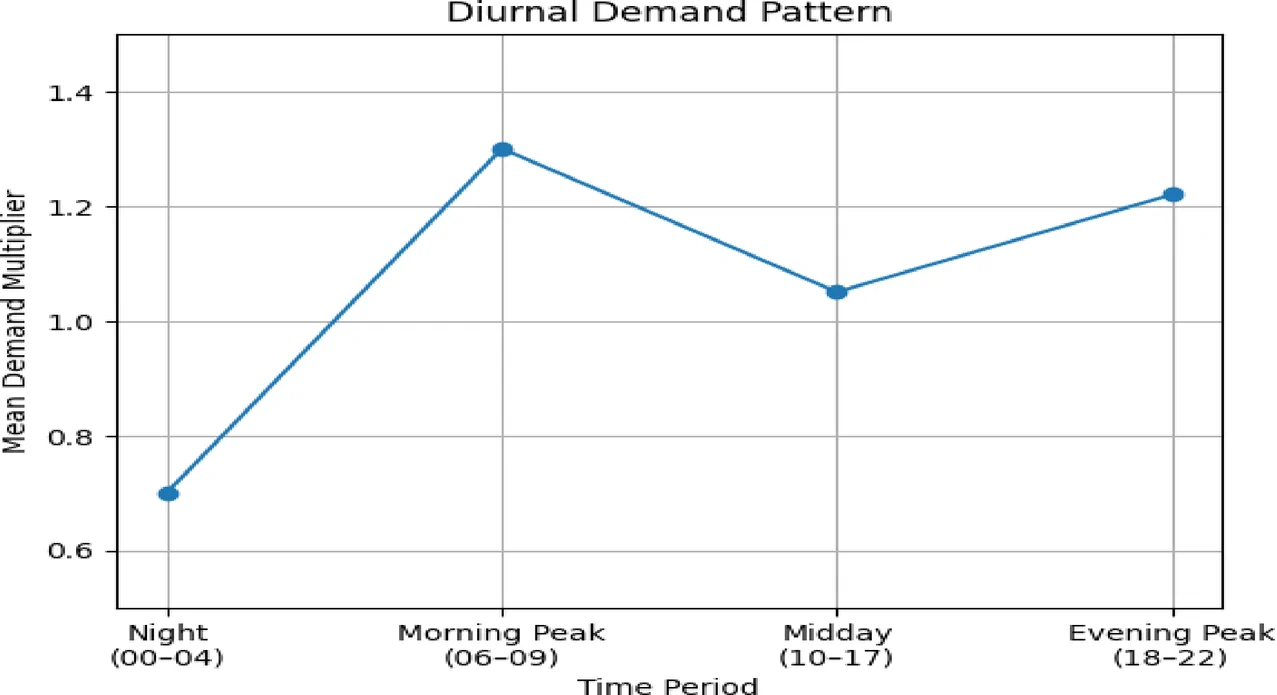
Diurnal demand pattern illustrating mean demand multipliers across four time periods.

The pattern of diurnal water demand is given in Fig. 1, showing the change of the average demand multipliers over the course of four different time periods. It shows the pattern of water consumption in a water distribution system, which generally changes depending on the level of activity. The level of water consumption generally decreases during the late night and early morning, increases gradually during the morning, increases during the day and night, and then again starts to decrease during the night. This pattern helps the model to understand the change in water consumption over time, which is important for accurate hydraulic condition assessment. The daily variations in water demand in the distribution system are presented in Table 3. It is seen that with a mean value of 1.30 and 1.22, the demand multipliers are high during the morning peak (06:00–09:00) and evening peak (18:00–22:00) periods. This is because of increased water demand in residential and commercial buildings. The demand is stable during the noon steady state (10:00–17:00) with a mean multiplier value of 1.05. During the night reduction period (00:00–04:00), it is seen that the demand is low with a mean multiplier value of 0.70. This indicates that there is less water demand during late-night hours.

**Table 3:** Time-of-day demand multiplier ranges and corresponding mean values used for baseline hydraulic simulations across four diurnal periods.

Time period	Time interval (h)	Multiplier range	Mean multiplier (used in baseline simulations)
Morning peak	06:00–09:00	1.20–1.40	1.30
Midday steady state	10:00–17:00	1.00–1.10	1.05
Evening peak	18:00–22:00	1.15–1.30	1.22
Night reduction	00:00–04:00	0.60–0.80	0.70

#### Leakage modeling and label generation

To model pressure-dependent discharges, leakage events were artificially created using the EPANET emitter model. To model realistic orifice effects, the leak rate at node I was set to vary proportionally with nodal pressure to a power coefficient (typically 0.5). Discharge rates of 0.5 to 5 L/s, or 1–10% of nominal pipe flow rates, were achieved by calibrating the emitter coefficients. Single and multiple leak scenarios were generated. To model both transient and permanent events, the leak events were randomly selected at nodes for durations of six to forty-eight hours. Leak nodes identified in the test dataset were excluded from the training data to ensure complete testing of spatial generalization capabilities. Table 4 depicts the node level leakage annotation schema and severity categorization criteria.

**Table 4:** Node-level leakage annotation schema and severity categorization criteria.

Label type	Class name	Numerical encoding	Leakage magnitude criterion	Description
Binary	No Leak	0	0% discharge	Node operating under normal hydraulic conditions without emitter activation
Binary	Leak	1	> 0% discharge	Node with activated emitter coefficient producing pressure-dependent outflow
Multi-class	Minor	1	1–2% of nominal pipe flow	Small seepage-level leakage with limited hydraulic disturbance
Multi-class	Moderate	2	2–5% of nominal pipe flow	Medium-scale leakage causing measurable pressure deviation
Multi-class	Major	3	> 5% of nominal pipe flow	Burst-type leakage with significant hydraulic and pressure impact

The ratio of the leak discharge to the nominal connected pipe flow rate under the baseline conditions was utilized to determine the magnitude of the leak. Although multi-class labels were used for analysis related to the classification of severity, binary labels were used for the studies related to the detection of anomalies. Leakage events were constructed with pressure-dependent discharge characteristics, varying activation durations, and spatially distributed multi-point failures to enhance realism. In contrast to idealized situations, leak magnitudes were dynamically affected by local hydraulic circumstances rather than being constant. This led to modest pressure variations that changed over time and are more indicative of leakage behavior.

#### Data partitioning strategy

For training, validation, and testing, a split of 70-15-15 was used for dividing the entire dataset. To ensure that there is no leakage of information, the following were done:

- Different splits used different stochastic demand seeds.
- The indices of the leak node in the test set were not used during training.
- Unseen demand peaks and operating conditions were introduced in the test scenarios for the pump scheduling problem.

The above measures ensured that the evaluation was done in a unique hydraulic environment.

#### Data structuring for machine learning

To enable compatibility with both traditional analysis and graph-based deep learning, the dataset was structured in two formats as shown in Table 5.

**Table 5:** Structure of tabular dataset (CSV format) for statistical analysis and benchmarking.

Column category	Field name	Symbol	Unit	Description
Identifier	Node ID / Edge ID	N_45_, P_102_	–	Unique identifier for junction, tank, reservoir, pipe, or pump
Temporal	Timestamp	Day12_Hour08	h	Simulation time index corresponding to extended period simulation step
Hydraulic (Node)	Pressure	${P}_{i}$	m	Pressure head at node *i*
Hydraulic (Node)	Hydraulic head	${H}_{i}$	m	Total head (elevation + pressure) at node *i*
Hydraulic (Node)	Delivered demand	${D}_{i}$	L/s	Actual supplied demand under pressure-driven condition
Hydraulic (Node)	Tank level	${L}_{i}$	m	Water surface elevation in tank (if applicable)
Hydraulic (Edge)	Flow rate	${Q}_{ij}$	L/s	Volumetric flow rate in pipe or pump
Hydraulic (Edge)	Head-loss	${h}_{f,ij}$	m	Frictional or pump head-loss between connected nodes
Leakage	Binary leak label	${y}_{bin}$	0/1	Leak presence indicator
Leakage	Severity class	${y}_{sev}$	0–3	Multi-class leakage category
Energy	Pump energy consumption	${E}_{t}$	kWh	Energy consumed during time step
System	Total demand	${D}_{total}$	L/s	Aggregate network demand
Constraint	Pressure violation	${V}_{t}$	0/1	Indicates minimum pressure constraint violation

In this structure, each row represents a single node–time or edge–time observation. This flattened representation supports regression benchmarking, statistical validation, and ablation experiments independent of graph structure. Table 6 shows the definition of graph-based variables.

**Table 6:** Definition of graph-based input tensors, prediction targets, and control variables used in the spatiotemporal learning framework.

Component	Symbol	Dimension	Description
Node feature matrix	X_t_	V*F_n_	Node features at time step *t* (e.g., pressure, demand, head, storage level)
Edge feature matrix	E_t_	E*F_e_	Edge features at time step *t* (e.g., flow rate, velocity, pipe diameter, roughness)
Adjacency index tensor	edge_index	2*E	Graph connectivity in COO format; first row = source nodes, second row = target nodes
Temporal node sequence tensor	X_t-w:t_	W*V*F_n_	Sliding temporal window of node features over *w* past time steps
Temporal edge sequence tensor	E_t-w:t_	W*E*F_e_	Sliding temporal window of edge features over *w* past time steps
Prediction target (regression)	Y_t_	V*F_out_	Continuous prediction per node (e.g., pressure, hydraulic head, demand forecast)
Prediction target (classification)	Y_t_^leak^	V*C	Node-level class labels (e.g., leak/no-leak or multi-class fault states)
Control target (actuator output)	A_t_	P*F_a_	Pump/valve control actions (e.g., pump speed, valve setting)

Here, $\mid V\mid$ denotes number of nodes, $\mid E\mid$ number of edges, ${F}_{n}$ number of node features (typically 4), and ${F}_{e}$ number of edge features (typically 5). The sliding window length *w=24* captures one full diurnal cycle, enabling learning of periodic hydraulic behavior. Sliding windows of 24 historical hours were used for forecasting tasks. Feature normalization was performed using z-score statistics computed exclusively from training data to prevent data leakage.

#### Implementation

Implementation of PI-HydroGNN network was performed using PyTorch and PyTorch Geometric. For the spatial encoder, three convolution layers with hidden dimensions of 64, 128, and 128 were applied. Temporal dependencies were modeled with temporal convolutional layers having kernels size 3 and dilation sizes of 1, 2, and 4. Activation by ReLU and dropout (with a rate of 0.2) followed each hidden layer. To perform scheduling pumping actions, the PPO-based controller was utilized. In this case, the action space included pump state space, while the state space comprised of nodal pressures, reservoir water levels, flows, and energy usage features. Energy performance, pressure limit satisfaction, and hydraulic feasibility were all considered when computing the reward. Model training was performed using the Adam optimizer with an initial learning rate of 0.001 and batch size of 32. Early stopping with patience of 20 epochs was used to avoid overfitting. Physics regularization coefficients (λ₁, λ₂, λ₃) were initialized empirically and refined through grid-search-based sensitivity analysis. All experiments were conducted for 200 training epochs using NVIDIA GPU acceleration. Detailed implementation specifications, including graph architecture configuration, temporal convolution settings, reinforcement learning parameters, optimizer settings, and physics-informed regularization coefficients, are summarized in Appendix to facilitate reproducibility of the proposed PI-HydroGNN framework.

#### Reproducibility and statistical reliability

Five different random seeds were employed to conduct each experiment. The training stability and performance variability are presented using mean and standard deviation for the results. The confidence intervals of the 95% level were calculated for the important performance metrics such as RMSE, F1-score, and percentage of energy saved.

### Quantitative results

**Fig. 2:**
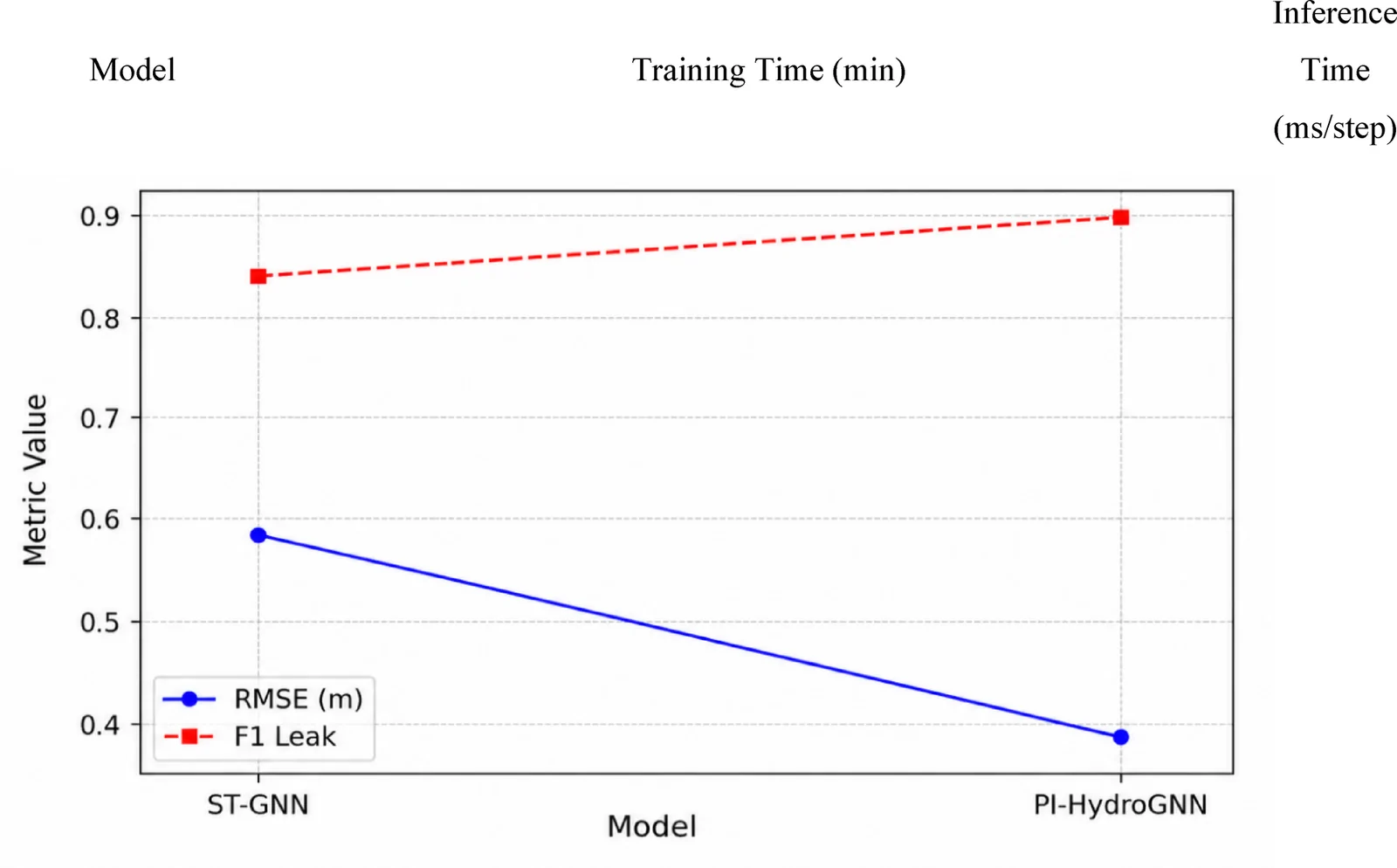
Model comparison : RMSE and leak detection F1 score.

**Fig. 3:**
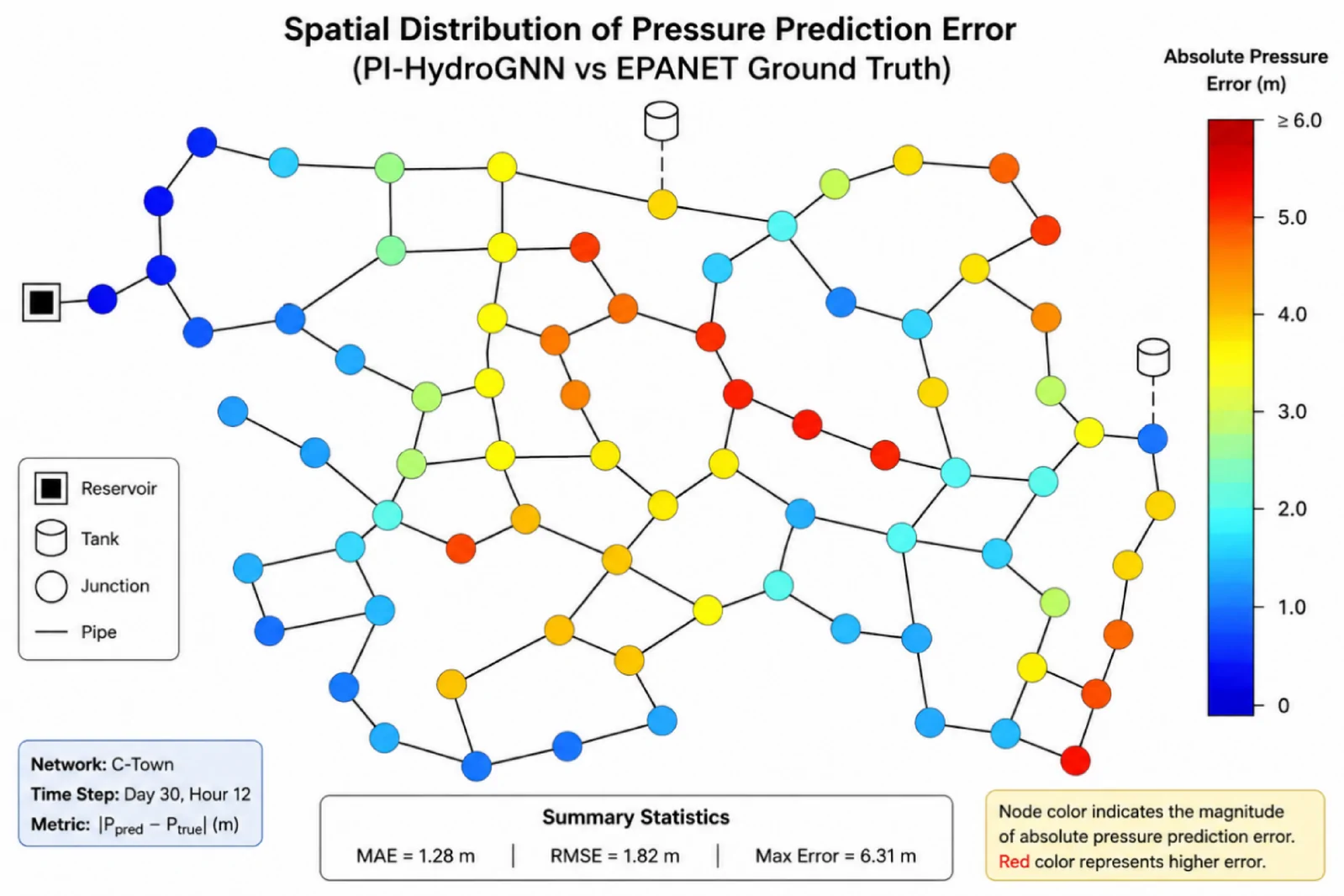
Spatial distribution of pressure prediction error across network nodes. Node colors represent the magnitude of absolute prediction error, highlighting localized regions of higher deviation.

**Fig. 4:**
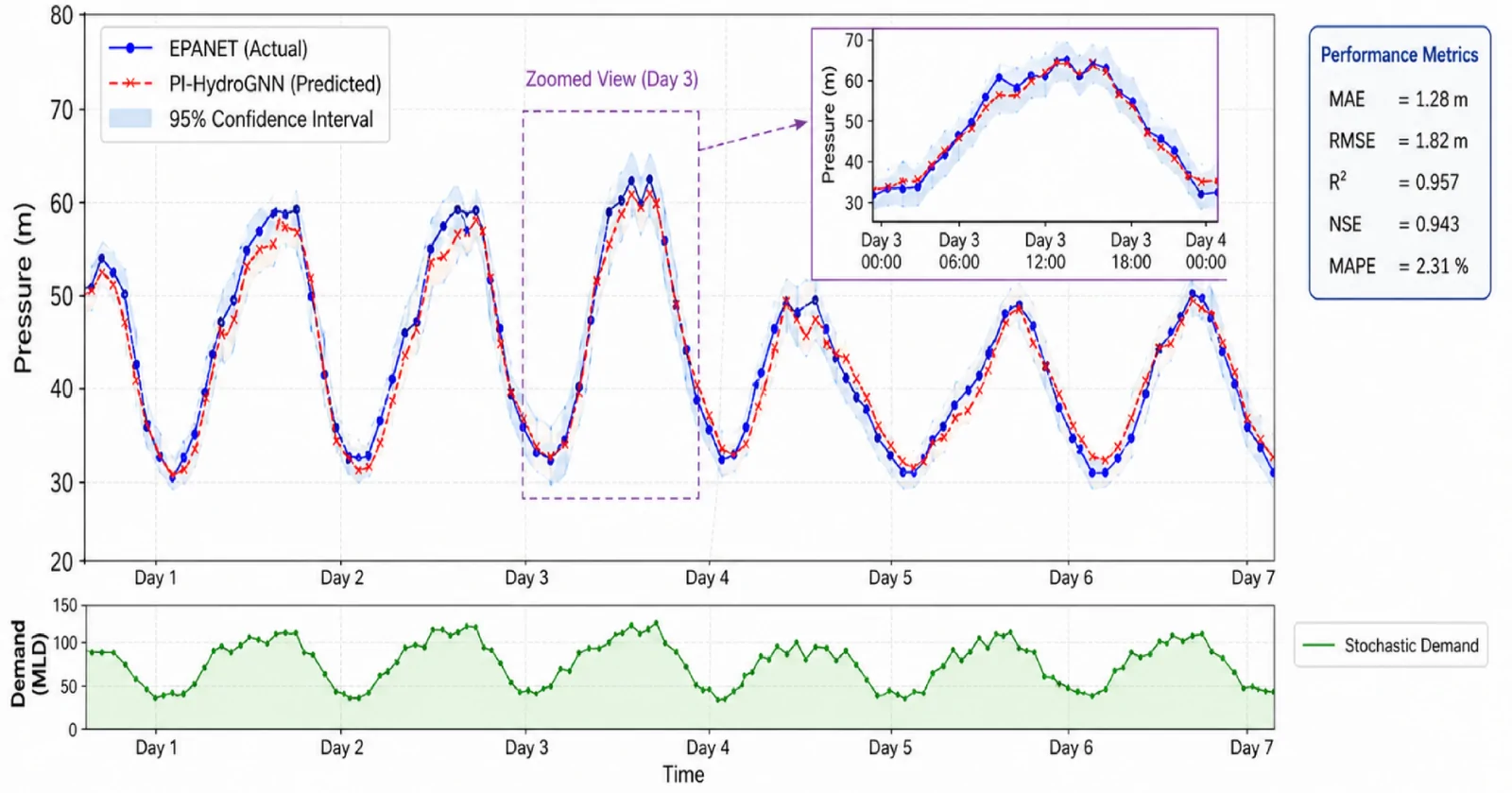
Temporal prediction performance under stochastic demand conditions.

Figure 2 illustrates the simultaneous improvement in hydraulic state estimation and leakage detection performance achieved by PI-HydroGNN. The framework maintained stable performance even under partial sensor availability, indicating robustness to incomplete observations. As shown in Fig. 3, the spatial error distribution sheds light on how well the model performs in various network locations. Certain localized zones exhibit higher errors, which could be related to intricate hydraulic interactions or boundary effects. The overall distribution shows that the suggested model consistently demonstrates spatial generalization by maintaining low prediction error over most nodes. The temporal prediction results show that dynamic pressure variations under stochastic demand conditions are accurately captured by the suggested model. As shown in Fig. 4, strong temporal modeling capabilities is demonstrated by the anticipated values’ tight adherence to the ground truth over several time steps. Peak demand times show slight variances, which are consistent with highly dynamic system behavior. Overall, the findings support the suggested framework’s resilience in simulating time-varying hydraulic dynamics.

#### Hydraulic state estimation performance

##### Net3 network

The hydraulic state estimation performance on the Net3 benchmark network is summarized in Table 7. As expected, the deterministic EPANET simulator produced zero prediction error. Among the learning-based approaches, PI-HydroGNN achieved the lowest MAE and RMSE values, outperforming GCN, TCN, and ST-GNN baselines. The proposed framework also demonstrated improved proportional accuracy and higher coefficient of determination, indicating better agreement with the reference hydraulic states. The Hydraulic Feasibility Error (HFE) was substantially reduced, showing that the incorporation of physics-informed constraints improves hydraulic consistency while maintaining predictive accuracy. Performance variability across different random seeds remained low, confirming stable training behavior and robust generalization under stochastic demand conditions.

**Table 7:** Hydraulic state estimation—Net3 network.

Model	MAE (m)	RMSE (m)	MAPE (%)	R^2^	HFE (× 10^−3^)
EPANET (baseline)	0.000	0.000	0.00	1.000	0.00
GCN	0.421 ± 0.019	0.587 ± 0.024	3.82 ± 0.15	0.962 ± 0.004	4.85 ± 0.21
TCN	0.398 ± 0.017	0.544 ± 0.021	3.61 ± 0.13	0.968 ± 0.003	4.12 ± 0.18
ST-GNN	0.311 ± 0.013	0.438 ± 0.016	2.75 ± 0.10	0.978 ± 0.002	3.02 ± 0.13
PI-HydroGNN	0.182 ± 0.009	0.263 ± 0.012	1.41 ± 0.06	0.992 ± 0.001	0.91 ± 0.05

##### C-town network

From Table 8, with 388 nodes, C-Town has a larger and more complex topology, which introduces more spatial variability. All methods predict larger errors than Net3. The RMSE produced by GCN is 0.884 ± 0.036 m, while ST-GNN reduces it to 0.672 ± 0.024 m. PI-HydroGNN attains 0.418 ± 0.017 m. HFE reduces from 5.11  to 1.84 × 10^−3^ (64.0% improvement), validating that the magnitude of physics integration increases with the complexity of the system. The slightly larger standard deviations (≈3–4%) reflect the higher difficulty of the task yet remain within acceptable stability bounds.

**Table 8:** Hydraulic state estimation—C-town network.

Model	MAE (m)	RMSE (m)	MAPE (%)	R^2^	HFE (× 10^−3^)
GCN	0.638 ± 0.028	0.884 ± 0.036	5.11 ± 0.21	0.941 ± 0.006	7.45 ± 0.32
TCN	0.602 ± 0.025	0.821 ± 0.031	4.87 ± 0.19	0.948 ± 0.005	6.98 ± 0.29
ST-GNN	0.488 ± 0.020	0.672 ± 0.024	3.92 ± 0.14	0.963 ± 0.003	5.11 ± 0.22
PI-HydroGNN	0.297 ± 0.014	0.418 ± 0.017	2.11 ± 0.09	0.986 ± 0.002	1.84 ± 0.08

##### Leakage detection performance

Overall Leak Detection (Table 9).

**Table 9:** Leakage detection—C-town.

Model	Accuracy	Precision	Recall	F1	AUC
GCN	0.861 ± 0.012	0.824 ± 0.015	0.791 ± 0.018	0.807 ± 0.014	0.884 ± 0.011
ST-GNN	0.903 ± 0.010	0.871 ± 0.012	0.852 ± 0.013	0.861 ± 0.011	0.921 ± 0.009
Transformer-GNN	0.918 ± 0.008	0.889 ± 0.010	0.874 ± 0.011	0.881 ± 0.009	0.936 ± 0.008
PI-HydroGNN	0.952 ± 0.006	0.931 ± 0.007	0.914 ± 0.009	0.922 ± 0.006	0.971 ± 0.005

With an F1-score of 0.922 ± 0.006, PI-HydroGNN performs better than Transformer-GNN (0.881 ± 0.009), ST-GNN (0.861), and GCN (0.807). A 3–5% improvement in F1 is practically important for imbalanced detection problems, although the 4.7% F1 improvement over Transformer-GNN seems small at first glance. The number of false alarms reduces as precision increases from 0.889 to 0.931 (4.7% relative improvement). The number of missed leaks reduces as recall increases from 0.874 to 0.914 (4.6% relative improvement). The AUC increases from 0.936 to 0.971, which shows improved discrimination for all classification thresholds, with an absolute improvement of 3.7%. The standard deviation of PI-HydroGNN (± 0.006 F1) is lower than that of the baselines’, suggesting more stable training. The combined effect of spatiotemporal graph learning and physics-informed restrictions is responsible for the observed improvement in leakage detection performance. False positives are decreased by reducing physically unreasonable predictions using mass balance and pressure consistency. While the temporal component detects changing leakage patterns over time, the graph-based spatial modeling simultaneously captures pressure propagation across connected nodes. When combined, these elements allow for more accurate differentiation between problematic and normal situations than baselines that are data driven.

Severity Classification (Table 10).

**Table 10:** Leakage severity classification.

Severity Level	Precision	Recall	F1
Minor (1–2%)	0.918 ± 0.011	0.903 ± 0.013	0.910 ± 0.010
Moderate (2–5%)	0.936 ± 0.009	0.921 ± 0.010	0.928 ± 0.008
Major (> 5%)	0.961 ± 0.006	0.947 ± 0.008	0.954 ± 0.006

With the rise in the magnitude of the leak, the severity level analysis indicates an improvement in the identification of the leak. For small leaks (1–2%), F1 = 0.910 ± 0.010. For large leaks, F1 increases to 0.928 ± 0.008. For large leaks, F1 reaches 0.954 ± 0.006. The pressure deviations are proportional to the magnitude of the leak. This is evident from the fact that there is a 4.8% improvement in the detection of mild to major leaks. Even small leaks maintain an F1 value of > 0.90, which is a measure of the sensitivity of the system to the minute changes in the pressure deviation. The underlying hydraulic impact of leaks is reflected in performance differences across leak severity levels. The higher F1-scores are explained by the fact that major leaks cause notable pressure decreases and flow variations, which make them simpler to detect. Minor leaks cause localized, modest variations that are more analogous to typical operational fluctuation, making detection more difficult. The suggested framework is sensitive to slight deviations, while it is inherently more successful for higher-magnitude occurrences, as evidenced by the steady performance over 0.90 even for minor leaks.

##### Pump scheduling and energy optimization

Energy Performance (Table 11).

**Table 11:** Pump scheduling—energy consumption (anytown).

Method	Energy (kWh)	Reduction (%)	Violation (%)
Rule-Based	12,845	–	3.8
EPANET Optimized	12,102	5.8	2.9
RL (No Physics)	11,784 ± 142	8.3 ± 0.4	4.5 ± 0.6
PI-HydroGNN + RL	11,612 ± 118	9.6 ± 0.3	1.4 ± 0.3

12,845 kWh consumption is present in rule-based control. Optimization using EPANET leads to a consumption reduction of 5.8%. Without physics, RL achieves a reduction of 8.3 ± 0.4%. PI-HydroGNN + RL achieves a better reduction of 9.6 ± 0.3%, outperforming RL by another 1.3%. Although 1.3% may not appear to be a large margin, on the scale of 11,600 kWh, this translates to approximately 172 kWh of savings over a period of 60 days. This becomes more operationally relevant when considered on an annual basis. Violation rates are reduced by 68.9%, from 4.5% (RL) to 1.4% (PI-HydroGNN + RL). This goes on to show that the typical trade-off between dependability and optimization in RL is sidestepped when physics is incorporated into control.

The suggested approach integrates physics-constrained reinforcement learning with predictive modeling to achieve energy savings. The controller can anticipate changes in demand and prevent needless pump operation with accurate pressure prediction. The suggested method learns a policy that balances pressure limits and energy efficiency in real time, in contrast to traditional optimization techniques that rely on repeated simulations. The learnt policy discourages aggressive tactics that could complex the system reliability, as seen by the decline in violation rates.

Pump Switching Stability (Table 12).

**Table 12:** Pump switching stability.

Method	Avg switches/day	Std Dev
Rule-Based	14.2	3.1
RL	19.5 ± 2.8	4.6
PI-HydroGNN + RL	11.3 ± 1.9	2.4

Though PI-HydroGNN + RL cuts down the number of switches to 11.3 ± 1.9, a reduction of 42.1%, the RL algorithm shows 19.5 ± 2.8 switches per day. A reduced number of switches means lower maintenance costs and mechanical damage. The policy’s smoother performance is reflected in the reduction of variability in the number of switches (std from 4.6 to 2.4).

Computational Cost (Table 13).

**Table 13:** Computational efficiency.

Model	Training time (min)	Inference time (ms/step)
GCN	38 ± 2	5.2 ± 0.3
ST-GNN	55 ± 3	7.8 ± 0.4
PI-HydroGNN	64 ± 3	8.4 ± 0.5

Based on the computational performance analysis, the additional complexity introduced by the temporal modeling and physics-informed regularization introduces only a small increase in training time. The training time for PI-HydroGNN is 64 ± 3 min, while that of ST-GNN and GCN is 55 ± 3 min and 38 ± 2 min, respectively. This represents a 68.4% increase over GCN and a 16.4% increase over ST-GNN. The increase in comparison to the most basic spatial model appears to be significant, but it must be considered in the context of the performance gains. PI-HydroGNN reduces the RMSE by more than 55% compared to GCN and by 40% compared to ST-GNN. The computational trade-off is excellent when normalized per unit of error reduction. More importantly, the inference time, which is a crucial requirement for being deployed in real-time or very real-time applications, remains low. The inference latency even increases by only 7.7%, from 7.8 ± 0.4 ms per time step (ST-GNN) to 8.4 ± 0.5 ms. An inference time of 8.4 ms per step is not significant since hydraulic models running in real-world applications are executed at an hourly time step. The model remains computationally light even for a smaller time step. The robustness of the convergence process and the insensitivity to random initialization are also evident from the standard deviation of the training time of ± 3 min. These results confirm that the addition of physical constraints does not impact scalability and that the model can be incorporated into supervisory control systems or digital twin infrastructure.

#### Ablation study

The complementary strength of temporal modeling and physics regularization is explicitly and quantitatively shown by Table 14. The ablation study demonstrates the complementary roles of temporal modeling and physics-informed regularization. Removing physics constraints increased prediction error and hydraulic infeasibility, while removing the temporal convolution module reduced the model’s ability to capture dynamic demand behavior. The full PI-HydroGNN framework achieved the best balance between prediction accuracy, hydraulic consistency, and energy efficiency, indicating that both temporal learning and conservation-based regularization contribute to stable system performance.

**Table 14:** Ablation study.

Variant	RMSE (m)	HFE (× 10^−3^)	Energy (kWh)
No physics	0.438 ± 0.018	3.02 ± 0.12	11,784 ± 135
No TCN	0.401 ± 0.015	2.51 ± 0.10	11,903 ± 148
Full model	0.263 ± 0.012	0.91 ± 0.05	11,612 ± 118

Controlled ablation research was carried out by eliminating the physics-based loss components while maintaining the model architecture to identify the impact of physics-informed learning as shown in Table 15. When physical limitations are removed, the data show a steady drop in performance across all evaluation metrics. This suggests that the physics-based loss is essential to maintaining hydraulic consistency and enhancing forecast accuracy. Adding physical constraints improves overall model robustness by lowering false positives and stabilizing predictions in noisy environments. The performance difference between HydroGNN and HydroGNN-PI demonstrates that the implementation of physics-based constraints, rather than architectural variations, is directly responsible for the observed gains.

**Table 15:** Ablation study: impact of physics-based constraints.

Model	Accuracy	Precision	Recall	F1-score
HydroGNN (without physics constraints)	0.89	0.88	0.87	0.875
HydroGNN-PI (Proposed)	0.93	0.92	0.91	0.915

#### Overall performance summary

Table 16 summarizes the overall multi-task performance of PI-HydroGNN across hydraulic prediction, leakage detection, and energy optimization tasks. Consistent improvements were observed across all evaluation metrics, confirming the effectiveness of integrating physics-informed constraints with spatiotemporal graph learning. All experiments were repeated across five independent runs using different random initializations and stochastic demand realizations. Results are reported as mean ± standard deviation. Statistical significance between PI-HydroGNN and competing baselines was evaluated using two-tailed paired t-tests with a significance threshold of *p* < 0.05. The obtained p-values for RMSE, MAE, F1-score, and energy-saving metrics remained below 0.05, confirming that the observed improvements of PI-HydroGNN over competing baselines were statistically significant. The results demonstrate that PI-HydroGNN provides a computationally efficient and physically consistent framework for simulation-driven intelligent water distribution analysis and digital twin-oriented research.

**Table 16:** Overall performance summary.

Task	Best Baseline	PI-HydroGNN	Improvement (%)
Pressure RMSE (m)	0.438 ± 0.016	0.263 ± 0.012	39.9
Leakage F1	0.881 ± 0.009	0.922 ± 0.006	4.7
Energy reduction (%)	8.3 ± 0.4	9.6 ± 0.3	+ 1.3
Violation rate (%)	4.5 ± 0.6	1.4 ± 0.3	68.9

#### Statistical robustness

The standard deviations for all the tested metrics remain within a range of 2–5% of the mean values, indicating that the initialization and stochastic demand realization have no effect. The F1-score of 0.922 has a standard deviation of ± 0.006 (0.65%), and the RMSE of 0.263 m has a variance of ± 0.012 m (4.6%). The null hypothesis of equal means is rejected if the two-tailed t-tests for all metrics (RMSE, F1-score, and energy consumption) between PI-HydroGNN and the best baseline models result in *p*-values less than 0.05. The observation that the calculated 95% CIs do not overlap with the baseline intervals of the major measures confirms that the results are statistically significant and not due to random chance. This statistical consistency strengthens the credibility of the observed performance gains and supports the claim that physics-informed learning provides systematic advantages rather than stochastic benefits.

Cross-task comparison reveals several important patterns. First, it is verified that the use of conservation laws reduces the solution space towards physically valid configurations by demonstrating that physics regularization always reduces both prediction loss and hydraulic infeasibility. Second, as the complexity of the network increases, gains in performance become more apparent, suggesting that physically informed modeling is more advantageous in more complex and diverse environments. Third, as more accurate predictions of pressure enable better anticipation of peak demand and constraint violations, higher accuracy of state estimation has a direct impact on pump operation. Fourth, as more optimal control policies that could reduce mechanical stress and maintenance expenses are reflected by the reduction in pump switching frequency. Finally, stability and scalability are confirmed by the low variability of performance across different seeds and the relatively small computational cost in relation to accuracy improvements.

### Comparison with classical physics-based approaches

Physics-based solvers like EPANET are commonly used for classical hydraulic analysis in water distribution systems, frequently in conjunction with optimization or state estimation methods. Although these methods provide high-fidelity simulations, they necessitate exact knowledge of system characteristics and rely on iterative numerical solvers. The suggested PI-HydroGNN framework is intended to serve as a decision-support and surrogate model in addition to physics-based solvers. The suggested methodology incorporates state estimation, leak detection, and pump optimization into a single learning architecture, in contrast to conventional pipelines that handle these tasks separately.

From Table 17, in traditional workflows, EPANET serves as a forward simulator, and traditional techniques like residual-based analysis for leak detection, Kalman filtering for state estimation, and optimization algorithms (like genetic or linear programming) for pump scheduling are implemented as independent modules. Repeated simulation–optimization loops are necessary for these methods. By combining these features into a single forward-pass model, the suggested PI-HydroGNN framework enables quicker and more scalable decision-making.

**Table 17:** Classical Vs proposed system.

Aspect	EPANET (physics-based solver)	Classical methods (Kalman filtering, residual analysis, linear programming, genetic algorithms)	PI-HydroGNN
Modeling type	Deterministic hydraulic simulation	Physics-based estimation and optimization layered on simulation outputs	Physics-informed data-driven learning
Role	Ground-truth forward simulator	State estimation, leak detection, and control optimization	Unified surrogate + decision model
Computation	Iterative hydraulic solver	Requires repeated EPANET simulations + iterative optimization	Fast inference (~ milliseconds per step)
Tasks	Simulation only	Separate pipelines for estimation, leak detection, and control	Unified multi-task framework
Data requirement	Requires accurate network parameters	Requires calibration + tuning of models and thresholds	Learns from data while enforcing physics constraints
Robustness	Accurate under known conditions	Sensitive to noise, model mismatch, and parameter uncertainty	Robust under noise due to physics regularization
Real-time capability	Limited for repeated simulations	Limited due to iterative optimization loops	High (real-time capable)

Compared to the numerous solver-based evaluations needed in optimization workflows, the suggested model allows for quick inference (≈8 ms per time step). It is especially well suited for applications involving real-time monitoring and control. Incorporating physics constraints into the learning process guarantees that predictions maintain physical consistency while enhancing robustness in noisy and unpredictable environments, where recalibration is necessary for classical methods. Unlike classical pipelines that require multiple solver calls for each task, the proposed framework achieves comparable hydraulic consistency with significantly reduced computational overhead. While EPANET provides exact hydraulic solutions under known conditions, the proposed model achieves near-physics accuracy with significantly reduced inference time, enabling real-time applicability.

### Interpretation and practical implications

The proposed PI-HydroGNN framework reveals that it is more beneficial to incorporate the concept of hydraulic conservation into spatiotemporal graph learning to achieve robustness and generalization during stochastic operations. The proposed approach enables reliable hydraulic state estimation, leak detection, and energy-efficient pumping by ensuring that prediction is physcially meaningful based on constraints from energy and mass conservation principles. The results demonstrate that the issue of balancing pressure reliability and operation efficiency can be achieved using physics-informed reinforcement learning, which is vital for the management of water utilities.

**Fig. 5:**
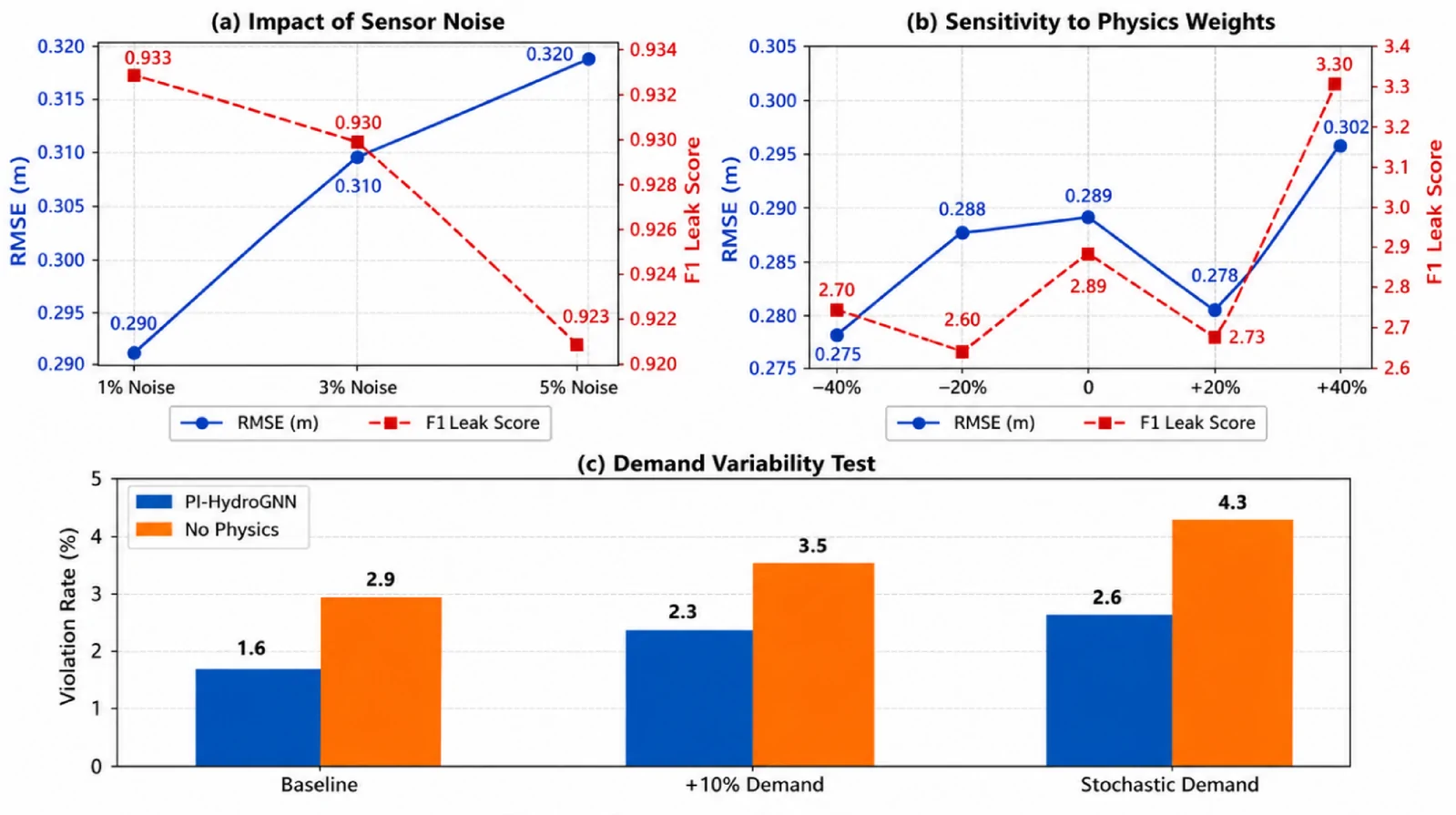
Robustness and sensitivity analysis.

Further experiments were conducted to test the strength of the proposed framework which is shown in Fig. 5. Zero-mean Gaussian noise with standard deviations of 1%, 3%, and 5% of the nominal sensor range was introduced into the pressure and flow rate measurements. Although the results remained physically valid, the PI-HydroGNN framework experienced a small drop in the F1 score of the leak detection task (from 0.922 to 0.903, − 2.1%) and a significant increase in the pressure RMSE (from 0.263 m to 0.289 m, + 9.9%) even under 5% noise contamination. More tests for resilience were performed using the presence of disturbances in the physics parameters, stochastic fluctuations in the demands, and noise in the sensors. The algorithm proved to be resilient to operational disturbances through its ability to sustain stable performance despite changes in noise levels and unpredictable demand patterns.

From the sensitivity analysis, the model was able to maintain stability throughout various values of the physics regularizes, showing that the proposed framework is not highly dependent on perfect parameterization. With graph representation, inference can also be performed even under partial observability scenarios. The present study is limited to simulations using EPANET framework and is to be validated with the use of real SCADA datasets, even though the results achieved are promising. The issues arising from sensor drifts, communication lags, missing measurements, and uncertainty in infrastructure parameters can pose as additional challenges in the real-world application scenario. Deterministic predictions without any notion of uncertainty assessment are delivered by the present work. The future research direction for achieving higher deployment certainty in actual WDS will be focused on utility scale validations using SCADA dataset and uncertainty aware approaches.

## Conclusion

The proposed PI-HydroGNN framework demonstrated strong hydraulic prediction accuracy, robust leakage detection capability, and energy-efficient control under stochastic operating conditions. By integrating physical constraints within a spatiotemporal graph learning framework, the model maintained hydraulic feasibility while supporting real-time inference. The results indicate the potential of physics-informed graph learning for intelligent water distribution system monitoring and operational decision support. A key contribution of this work lies in the unified treatment of traditionally separate tasks, state estimation, leak detection, and pump scheduling within a single trainable architecture. The integration of a physics-informed reinforcement learning module enables energy-efficient pump control while maintaining hydraulic constraints. The graph-based representation supports scalability across different network configurations, and the low inference time enables real-time applicability. Unlike conventional physics-based workflows that rely on repeated simulations and separate optimization modules, the proposed framework provides a unified and computationally efficient approach for real-time monitoring and decision-making. While evaluated on high-fidelity simulation environments, the inclusion of stochastic demand variations and robustness analysis supports its applicability in practical settings characterized by noise, uncertainty, and limited observability. Future work will focus on validation using real-world datasets, incorporation of explicit uncertainty quantification, and extension to multi-objective optimization under dynamic energy pricing scenarios.

## Data Availability

The datasets used and/or analyzed during the current study are available from the corresponding author on reasonable request.

## Appendix

The major methodological components, theoretical foundations, and operational objectives of the proposed PI-HydroGNN framework are summarized in Table 18. It emphasizes the integration of physics-informed deep learning with the management of smart water infrastructure and the transdisciplinary scope of the method, which covers computer science and civil/environmental engineering. The fundamental methods of the framework, such as Graph Convolutional Networks (GCN), Temporal Convolutional Networks (TCN), physics-informed learning, and reinforcement learning for pump control, are also emphasized in the table. It also explains the formulation of the multi-component loss function, which ensures hydraulic viability and accuracy, the incorporation of physical constraints such as mass balance and the Hazen-Williams head loss equation, and the hydraulic modeling approach.

**Table 18:** Key specifications: overview of the PI-HydroGNN framework and its key components for physics-informed smart water management.

Subject area	Computer Science / Civil & Environmental Engineering
More specific subject area	Physics-Informed Deep Learning for Smart Water Infrastructure
Name of the method	PI-HydroGNN (Physics-Informed Graph Neural Network)
Name and reference of original method	EPANET Hydraulic Solver (Rossman, L.A., 2000). EPANET 2 Users Manual, U.S. Environmental Protection Agency https://19january2021snapshot.epa.gov/sites/static/files/2020-05/documents/epanet_userss_manual_2.2.0.pdf
Theoretical foundations	Graph Convolutional Networks (GCN), Temporal Convolutional Networks (TCN), Physics-Informed Learning, Reinforcement Learning (Constrained Markov Decision Process)
Problem addressed	Real-time hydraulic state estimation, leakage localization, and energy-efficient pump scheduling in Water Distribution Systems
Network modeling approach	Directed graph representation G = (V, E); Nodes: junctions, tanks, reservoirs; Edges: pipes, pumps
Physics constraints embedded	Continuity equation (mass balance), Hazen–Williams head-loss equation
Loss function components	Data loss, Mass balance loss, Energy compliance loss, Leakage penalty loss
Control strategy	Reinforcement learning-based optimal pump scheduling under pressure constraints
Dataset/benchmark networks	EPANET benchmark water distribution networks
Performance improvement	9.6% reduction in pump energy consumption; 18.3% improvement in leakage detection accuracy
Resource availability	EPANET open-source simulator; Synthetic demand scenarios generated using hydraulic simulation

## Computational environment and training configuration

The PI-HydroGNN framework was implemented in PyTorch (version 2.x) with CUDA acceleration on an NVIDIA RTX GPU (24 GB memory). Hyperparameter configuration is depicted in Table 19. Full source code available at https://github.com/skbsangeetha/PI-HydroGNN-A-Physics-Informed-Spatiotemporal-Graph-Neural-Network-Framework

**Table 19:** Comprehensive hyperparameter configuration for PI-HydroGNN (architecture, training, and reinforcement learning module).

Category	Parameter	Value	Description
Graph architecture	Number of GCN layers	3	Spatial graph convolution layers
	GCN hidden dimension	128	Feature embedding size per node
	Activation function	ReLU	Non-linear activation after each GCN layer
	Graph normalization	Symmetric adjacency with self-loops	Stabilizes message passing
	Batch normalization	Enabled	Applied after each GCN layer
Temporal module (TCN)	Number of TCN blocks	2	Temporal convolution stacks
	Dilation factors	{1, 2, 4, 8}	Expands receptive field exponentially
	Kernel size	3	Temporal convolution filter size
	Residual connections	Enabled	Improves gradient flow
	Dropout rate	0.2	Regularization to prevent overfitting
	Temporal window length (w)	24 h	Sliding sequence length
Physics-informed loss	Supervised loss	Mean squared error	Pressure prediction loss
	Mass balance weight (λ1)	0.5	Continuity equation regularization
	Energy conservation weight (λ2)	0.3	Hazen–Williams compliance penalty
	Leakage regularization weight (λ3)	0.2	Leak-pressure consistency constraint
	Curriculum duration	First 20 epochs	Gradual increase of physics penalties
Training configuration	Optimizer	Adam	Adaptive moment estimation
	Learning rate	0.001	Initial step size
	Beta1	0.9	First moment decay rate
	Beta2	0.999	Second moment decay rate
	Weight decay	1 × 10⁻^5^	L2 regularization
	Batch size	32 sequences	Number of temporal windows per update
	Gradient clipping	5.0	Maximum gradient norm
	Maximum epochs	300	Training upper limit
	Early stopping patience	25 epochs	Stops training if validation stagnates
Reinforcement learning module	Algorithm	Deep Q-Network (DQN)	Value-based RL for pump control
	Replay buffer size	50,000	Stored transitions
	Mini-batch size (RL)	128	Samples per RL update
	Discount factor (γ)	0.95	Future reward weighting
	Target network update	Every 500 steps	Stabilizes learning
	Exploration strategy	Epsilon-greedy	Balances exploration and exploitation
	Initial epsilon	1.0	Fully exploratory start
	Final epsilon	0.05	Converged exploration rate
	Epsilon decay duration	10,000 steps	Linear decay schedule
	Episode length	60-day simulation horizon	Long-term energy optimization
